# Autoimmune encephalitis in COVID-19 patients: a systematic review of case reports and case series

**DOI:** 10.3389/fneur.2023.1207883

**Published:** 2023-09-13

**Authors:** Hua Xue, Li Zeng, Hongxian He, Dongxun Xu, Kaixin Ren

**Affiliations:** ^1^Department of Neurology, Sichuan Taikang Hospital, Chengdu, Sichuan, China; ^2^Department of Respiratory, Affiliated Hospital of Youjiang Medical University for Nationalities, Baise, Guangxi, China; ^3^Department of Rehabilitation, Affiliated Hospital of Yunnan University, Kunming, Yunnan, China

**Keywords:** COVID-19, autoimmune encephalitis, SARS-CoV-2, systematic review, anti-N-methyl-D-aspartate encephalitis

## Abstract

**Background:**

There is mounting evidence suggesting that autoimmune encephalitis (AE) can be observed as a neurological complication in patients with COVID-19. This review aimed to summarize the clinical manifestations, types, and outcomes of COVID-19-associated AE.

**Methods:**

A systematic search was conducted in the PubMed, Embase, and Web of Science databases to identify case reports and case series related to COVID-19-associated AE from 1 January 2020 to 31 March 2023. After a thorough screening and evaluation, irrelevant articles were excluded. Relevant information concerning types, clinical manifestations, and outcomes was extracted and synthesized.

**Results:**

A total of 37 studies, comprising 34 case reports and 3 case series, were included in this review. Among the 42 COVID-19-associated AE patients, 21 (50%) cases were classified as an unknown antibodies (Ab) type of COVID-19-associated AE, 10 (23.80%) cases as anti-N-methyl-D-aspartate (NMDA) encephalitis, 4 (9.5%) cases as limbic encephalitis, and 3 (7.1%) cases as anti-myelin-oligodendrocyte-glycoprotein encephalitis, along with other rare types of AE. Disturbance of consciousness, seizures, and psychiatric symptoms were identified as the main clinical manifestations of COVID-19-associated AE. While the symptoms of AE displayed variation, most patients achieved full recovery although a few experienced residual symptoms of neurological damage.

**Conclusion:**

This systematic review comprehensively describes the characteristics of COVID-19-associated AE. The main type of COVID-19-associated AE identified in this study is an unknown Ab type of COVID-19-associated AE. Despite the potentially life-threatening risks of COVID-19-associated AE, the majority of patients survived, with some patients reporting residual neurological symptoms.

## 1. Introduction

Coronavirus disease 2019 (COVID-19) is an emerging infectious disease caused by a severe acute respiratory syndrome coronavirus 2 (SARS-CoV-2) infection (1). It has placed an unprecedented burden on economies and healthcare systems worldwide since the beginning of the COVID-19 pandemic (2). Recent studies have demonstrated that both COVID-19 patients and those with autoimmune diseases exhibit similar immune responses affecting multiple organs and systems, including the respiratory, neurological, cardiovascular, hematologic, and endocrine systems (3). Overproduction of cytokines and overactivation of immune cells by the immune system lead to uncontrolled immune reactions resulting in organ damage and the production of autoantibodies due to the breakdown of immune tolerance (3). Additionally, molecular mimicry triggered by SARS-CoV-2 infection may induce autoimmunity in COVID-19 patients (4).

During the initial stages of the COVID-19 outbreak, several studies indicated that 36.4% of patients experienced neurological symptoms, affecting various parts of the nervous system such as the central nervous system, peripheral nervous system, and skeletal muscles (5). Furthermore, COVID-19 exerts a significant impact on the neuropsychological health of both patients and caregivers. Olfactory and gustatory deficits are considered the most common manifestations of peripheral nerve damage in COVID-19 patients, with incidence rates as high as 41.0 and 38.2%, respectively (6, 7). Peripheral nervous system involvement can also lead to symptoms such as facial nerve palsy and oculomotor abnormalities in COVID-19 patients (7, 8). A study of mental and psychological burdens in 204 countries estimated that the global prevalence of depressive disorders increased by 28% and anxiety disorders by 26% during the COVID-19 pandemic (9).

The neurological impacts of COVID-19 extend beyond dizziness, headaches, facial paralysis, and strokes. A mounting body of evidence has documented cases of COVID-19-associated autoimmune encephalitis (AE) (10). Initially, some symptoms may appear mild, such as fever, headache, dizziness, and lethargy, which could be easily overlooked or mistaken for medication side effects or other unspecified infections (4). However, as the disease progresses, more severe symptoms may manifest, including mental disorders, abnormal behavior, amnesia, aphasia, clumsiness, convulsions, and, in severe cases, loss of consciousness and coma (11). In a meta-analysis conducted by Siow et al., which encompassed 129,008 patients with COVID-19, the prevalence of encephalitis was found to be 0.215% (11).

COVID-19 patients, especially in severe cases, exhibited abnormal inflammatory responses that led to hyperactive innate immune reactions, excessive inflammation, and increased levels of pro-inflammatory mediators, cytokines, chemokines, ferritin, and D-dimer (12). Lower-than-normal levels of helper T cells and suppressor T cells in severe COVID-19 patients suggest possible lymphocyte damage and the induction of adaptive immune hyperfunction by SARS-CoV-2 (13). Lymphocytes, which express angiotensin-converting enzyme 2 (ACE-2), serve as a binding site for SARS-CoV-2, leading to lymphocyte death and COVID-19-associated AE (14). The mechanisms by which SARS-CoV-2 enters the brain and causes encephalopathy are still a matter of controversy, and several major theories have been proposed. First, it is possible that SARS-CoV-2 may be transmitted retrogradely through axons in the olfactory, respiratory, and enteric nervous system networks (14). Second, it is suggested that SARS-CoV-2 can breach the blood-brain barrier by disrupting the endothelial lining, thereby gaining access to brain tissue (15, 16). Once inside the brain, SARS-CoV-2 can interact with both neuronal and non-neuronal cells that express ACE-2 receptors (17, 18).

Despite accumulating reports of COVID-19-associated AE, few systematic reviews have summarized AE as a neurological complication of COVID-19. To gain a better understanding of COVID-19-associated AE, we performed a comprehensive assessment of the clinical manifestations, types, and outcomes of COVID-19-associated AE.

## 2. Methods

This review was conducted in accordance with the guidelines for preferred reporting elements for systematic reviews and meta-analyses (PRISMA) (19).

### 2.1. Search strategy

We conducted an extensive and exhaustive search of case reports and series between 1 January 2020 and 31 March 2023 from PubMed, Embase, and Web of Science. The search terms included “Autoimmune Encephalitis,” “Antibody-Mediated Encephalitis,” “Antibody Mediated Encephalitis,” “limbic encephalitis,” “Anti NMDA,” “Anti MOG,” “Anti LGI1,” “Anti GABA,” “coronavirus disease-19,” “SARS-CoV-2,” “COVID 19,” and “COVID-19.” All English publications describing case reports and case series of confirmed COVID-19 infections with AE were included. Two independent reviewers comprehensively searched and filtered the eligible studies. We excluded all review articles, other types of encephalitis, and unconfirmed cases of AE.

### 2.2. Inclusion criteria

This case report and case series met the following inclusion criteria: (1) patients tested positive for SARS-CoV-2 in the reverse transcription-polymerase chain reaction (RT-PCR) or had a positive title for SARS-CoV-2 serum antibody and (2) raw data on clinical symptoms, diagnosis, treatment, and outcome of COVID-19-associated AE were provided.

### 2.3. Study selection and data extraction

Two reviewers independently selected studies by reading the full articles and then extracting data from the eligible articles. The following data were extracted from the included studies: the first author's last name, year of publication, number of patients, participant's age, participant gender, SARS-CoV-2 diagnosis method, neurological manifestations, imaging analysis, cerebrospinal fluid (CSF), types, AE diagnosis method, treatment details, and outcomes.

## 3. Results

### 3.1. Searching published literature

**Figure 1:**
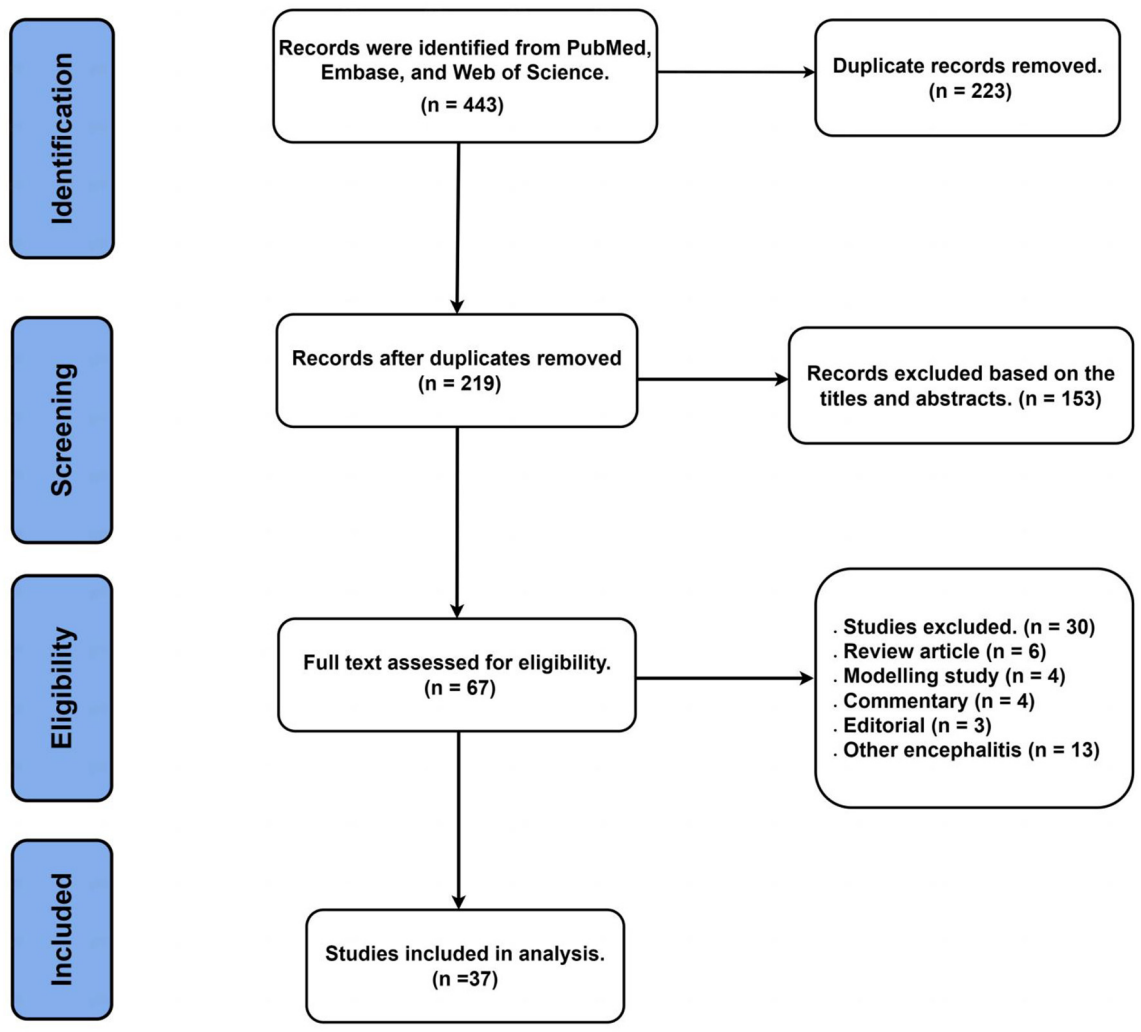
Flowchart of the trial selection process for this systematic review.

A total of 443 articles were yielded after a comprehensive search in our literature. After removing 223 duplicate articles, 153 irrelevant title and abstract articles and 66 full-text articles were further evaluated. Finally, 37 studies were included in our systematic review after excluding 6 review articles, 4 modeling studies, 4 commentary, 3 editorials, and 13 other encephalitides (including herpes simplex virus encephalitis, cytomegalovirus encephalitis, and varicella-zoster virus encephalitis) (Figure 1) (20–56).

### 3.2. Study characteristics

This article includes 37 studies, consisting of 34 case report studies and 3 case series. The basic information of the included studies is shown in Table 1. A total of 42 participants were included in this systematic review. The case reports and case series were conducted in various countries, including the United States (*n* = 17), Italy (*n* = 4), Iran (*n* = 2), the United Kingdom (*n* = 2), Spain (*n* = 2), Korea (*n* = 2), India (*n* = 2), Singapore (*n* = 1), France (*n* = 1), Egypt (*n* = 1), Mexico (*n* = 1), Germany (*n* = 1), Japan (*n* = 2), Turkey (*n* = 1), and China (*n* = 1).

**Table 1:** Characteristics of included studies for autoimmune encephalitis as a neurological complication in COVID-19 patients.

**No.**	**References (Location)**	**Number of patients**	**Sex**	**Age (years)**	**SARS-CoV-2 diagnosis method**	**The onset time of AE**	**Neurologicalmanifestations**	**Imaging analysis**	**CSF analysis**	**Type of AE**	**AE diagnosismethod**	**Medical history**	**Treatment**	**Outcome**
1	Sarigecili et al. (20) (Turkey)	1	M	7	RT-PCR	NM	Ataxia, wide-based gait, somnolence, seizures, choreiform movements in the hands and feet.	aMRI: unremarkable	Protein, glucose, lactate, pyruvate, IgG, EBV, HSV-1, HCV-2: normal. Anti-NMDA: positive.	Anti-NMDA encephalitis.	CSF analysis	None	Methylprednisolone, IVIG, symptomatic treatment, etc.	Complete recovery
2	Kaur et al. (21) (India)	1	M	1	RT-PCR	5 days	Seizures, peri-oral dyskinesias, bilateral striatal toe, extrapyramidal movements.	aMRI: unremarkable	Protein: 27 mg/dl, glucose: 65 mg/dl. Anti-NMDA: positive.	Anti-NMDA encephalitis.	CSF analysis	None	Methylprednisolone, IVIG, rituximab, symptomatic treatment, etc.	Complete recovery
3	Burr et al. (22) (USA)	1	F	23	RT-PCR	NM	Seizures.	aMRI: unremarkable	Protein: 25 mg/dl, glucose: 65 mg/dl. Anti-NMDA: positive.	Anti-NMDA encephalitis.	CSF analysis	None	Methylprednisolone, IVIG, symptomatic treatment, etc.	Complete recovery
4	Scheuermeier et al. (23) (Italy)	1	F	4	RT-PCR	NM	Generalized tonic-clonic seizures, cognitive deterioration.	aMRI: hyperintense lesions in the bilateral insular cortex and medial temporal region.	Protein, glucose, cells lactate: normal. Anti-NMDA was negative in CSF but positive in serum.	Anti-NMDA encephalitis.	CSF analysis, brain MRI	None	Benzodiazepines, plasmapheresis, symptomatic treatment, etc.	Partial recovery
5	Chakraborty et al. (24) (USA)	1	M	61	RT-PCR	5 days	Abnormal behavior, drowsiness, aphasia.	aMRI: hyperintense lesions in left temporoparietal regions.	Protein: 66 mg/dl, anti-NMDA: negative, SARS-CoV-2 IgG and spike protein antibodies: positive.	Unknown Ab type of COVID-19-associated AE.	CSF analysis, brain MRI	Hypertension, diabetes	Methylprednisolone, symptomatic treatment, etc.	Complete recovery
6	Jeanneret et al. (25) (USA)	1	M	21	RT-PCR	NM	Gait instability, dysarthria, generalized tonic-clonic seizures.	aMRI: hyperintense lesions in brainstem and cerebellum.	Zero white blood cells, protein count 122 mg/dl, normal glucose, zero oligoclonal bands, and normal IgG index.	Unknown Ab type of COVID-19-associated AE.	CSF analysis, brain MRI	Diabetes	IVIG, plasmapheresis, levetiracetam.	Partial recovery
7	Hosseini et al. (26) (USA)	2	➀ M ➁ F	➀ 46 ➁ 79	RT-PCR	➀ 2 days ➁ 2 days	➀ Delirium, disinhibition, confusion, status epilepticus. ➁ Generalized seizures	➀ aMRI: three hyperintense foci. ➁ aMRI: limbic system with partial diffusion restriction	➀ Mildly raised protein and oligoclonal bands. ➁ Protein, glucose, cells lactate: normal.	➀ Unknown Ab type of COVID-19-associated AE. ➁ Limbic encephalitis.	CSF analysis, brain MRI	➀ None ➁ None	➀ Ceftriaxone, aciclovir, and sodium valproate ➁ Levetiracetam	Complete recovery
8	Hilado et al. (27) (USA)	3	➀ M ➁ M ➂ M	➀ 6 ➁ 12 ➂ 12	➀ Positive SARS-CoV-2 serum antibody ➁ Positive SARS-CoV-2 serum antibody ➂ RT-PCR	➀ 12 days ➁ 2 days ➂ 5 days	➀ Headache, urinary retention, lower extremity weakness, and ataxic gait. ➁ Generalized seizures. ➂ Seizures.	➀ aMRI: hyperintense lesions in bilateral parietal. ➁ aMRI: unremarkable. ➂ eMRI: bilateral frontoparietal lobes with diffuse leptomeningeal enhancement concerning.	➀ Anti-SARS-CoV-2, anti-NMDA: negative ➁ Anti-SARS-CoV-2, anti-NMDA: negative; protein: mild high. ➂ Anti-SARS-CoV-2, anti-NMDA: negative.	Unknown Ab type of COVID-19-associated AE.	➀ Brain MRI, CSF analysis. ➁ CSF analysis. ➂ Brain MRI.	➀None ➁Autism spectrum disorders ➀Migraine	➀ Methylprednisolone, symptomatic treatment, etc. ➁ Levetiracetam, symptomatic treatment, etc. ➂ Methylprednisolone, symptomatic treatment, etc.	➀ Complete recovery ➁ Complete recovery ➂ Complete recovery
9	Álvarez et al. (28) (USA)	1	F	30	RT-PCR	NM	Focal seizures and some generalized seizures.	aMRI: hyperintense lesions in left hippocampus.	Protein: 54.5 mg/dl; predominantly lymphocytic (90%) leukocyte count of 44 cells/L. Anti-NMDA: positive.	Anti-NMDA encephalitis.	Brain MRI, CSF analysis	None	Methylprednisolone, symptomatic treatment, etc.	Partial recovery
10	Farhadian et al. (29) (USA)	1	F	78	RT-PCR	2 days	Seizure, confusion, and disorientation	aMRI: atrophy and patchy periventricular and subcortical white matter hyperintensities.	350 red cells/μl, 1 white blood cell/μl, 75% lymphocytes, 25% monocytes, glucose 67 mg/dl and protein 43 mg/dl.	Unknown Ab type of COVID-19-associated AE.	Brain MRI, CSF analysis	Kidney transplant	Hydroxychloroquine, tocilizumab.	Complete recovery
11	Mekheal et al. (30) (USA)	1	F	88	Positive SARS-CoV-2 serum antibody.	3 days	Leg weakness and dysarthria.	eMRI: evidence of an old infarct. CT brain: intraparenchymal hemorrhage.	Protein: 145 mg/dl; red cells: 53.	Unknown Ab type of COVID-19-associated AE.	CSF analysis	Hypertension	Methylprednisolone, IVIG, symptomatic treatment, etc.	Death
12	Kato et al. (31) (Japan)	1	F	55	RT-PCR	8 days	Consciousness disturbance, myoclonic-like movements and gait disturbance	Not reported	Glucose: 65 mg/dl; protein: 59 mg/dl.	Unknown Ab type of COVID-19-associated AE.	CSF analysis	None	Methylprednisolone, symptomatic treatment, etc.	Complete recovery
13	Panariello et al. (32) (Italy)	1	M	23	Chest CT RT-PCR	NM	Psychomotor agitation, anxiety, persecutory delusions and auditory hallucinations, and insomnia.	CT brain: unremarkable	Protein, glucose, cells lactate: normal. Anti-NMDA: positive.	Anti-NMDA encephalitis.	CSF analysis	None	IVIG, symptomatic treatment, etc.	Complete recovery
14	Monti et al. (33) (Italy)	1	M	50	RT-PCR	NM	Refractory status epilepticus	aMRI: unremarkable	Eurotropic fungi, bacteria and viruses: negative. Anti-NMDA: positive.	Anti-NMDA encephalitis.	CSF analysis	Hypertension	Metilprednisolone, IGIV, plasma-exchange, symptomatic treatment, etc.	Complete recovery
15	Dono et al. (34) (Italy)	1	M	81	Chest CT RT-PCR	7 days	Mild confusion with fluctuation of the mental status.	aMRI: hyperintense lesions in the bilateral parietal cortex, left temporal cortex, and right cingulate cortex.	Lymphocytosis (26 cell/mm^3^), normal glucose level (78 mg/dl), slight protein increase (47 mg/dl), and positive oligoclonal bands.	Unknown Ab type of COVID-19-associated AE.	Brain MRI, CSF analysis	Hypertension	Methylprednisolone, IVIG, symptomatic treatment, etc.	Death
16	Moriguchi et al. (35) (Japan)	1	M	24	Chest CT	9 days	Headache, consciousness disturbance, seizures.	aMRI: hyperintense lesions in the right mesial temporal lobe and hippocampus.	Glucose, HSV-1, HSV-2, HHV-6, HHV-7: normal. anti-SARS-CoV-2: positive.	Limbic encephalitis.	Brain MRI, CSF analysis	None	Steroids, symptomatic treatment, etc.	Complete recovery
17	Jacobs et al. (36) (USA)	1	M	65	RT-PCR	NM	Vertigo, non-specific blurry vision, assaultive behavior, agitated, combative, and hallucinatory.	aMRI: four ring-shaped postcontrast enhanced lesions	Protein: 52 mg/dl. Detailed, extensive infection: negative. Anti-MOG IgG: positive.	Anti-MOG encephalitis.	Brain MRI, CSF analysis	None	Methylprednisolone, IVIG, symptomatic treatment, etc.	Complete recovery
18	Raynowska et al. (37) (USA)	1	M	31	RT-PCR	NM	Progressively worsening confusion, forgetfulness, visual hallucinations, dysphagia, and dysarthria.	aMRI: unremarkable,	Protein, glucose, cell: normal. Anti-AMPA and Anti-CRMP positive.	Anti-AMPA combined anti-CRMP-5 encephalitis.	CSF analysis.	Myasthenia gravis	IVIG, plasmapheresis, symptomatic treatment, etc.	Not reported.
19	Durovic et al. (38) (Germany)	1	M	22	RT-PCR	NM	Severe headache, mild impairment in executive functions.	aMRI: multiple disseminated hyperintensities, predominantly cortically	Protein: 39.9 mg/dl; glucose: 64 mg/dl; HSV-1, HSV-2: normal. MOG IgG: positive.	Anti-MOG encephalitis	Brain MRI, CSF analysis.	None	Methylprednisolone, symptomatic treatment, etc.	Complete recovery
20	Koh et al. (39) (Korea)	1	F	20	Chest CT RT-PCR	NM	Focal tonic-clonic seizure	aMRI: diffuse cortical high signal intensities.	Protein, glucose, cells lactate, HSV-1: normal.	Unknown Ab type of COVID-19-associated AE.	Brain MRI.	None	Methylprednisolone, IVIG, symptomatic treatment, etc.	Complete recovery
21	Valadez-Calderon et al. (40) (Mexico)	1	M	28	RT-PCR	2 weeks	Altered mental state characterized by incoherent speech, somnolence, auditory hallucinations, suicidal ideation, and generalized tonic-clonic seizures.	aMRI: hyperintensities in the bilateral anterior cingulate cortex and temporal lobes.	Anti-SARS-CoV-2: negative. Anti-NMDAR and anti-GAD65: positive.	Anti-NMDA combined anti-GAD65 encephalitis.	Brain MRI, CSF analysis.	None	Methylprednisolone, symptomatic treatment, etc.	Not reported.
22	Dahshan and Abdellatef (41) (Egypt)	1	M	67	RT-PCR	8 days	Confusion, behavioral changes, agitation, and one attack of loss of consciousness.	aMRI: unremarkable	Normal cell count, normal proteins, glucose, LDH, and chloride. Anti-SARS-CoV-2: positive.	Unknown Ab type of COVID-19-associated AE.	CSF analysis.	Hypertension	Methylprednisolone, symptomatic treatment, etc.	Complete recovery
23	Peters et al. (42) (USA)	1	M	23	RT-PCR	5 weeks	Cognitive slowing, personality changes, generalized seizures.	aMRI: hyperintensities in diffuse left hemispheric cortical	HSV, VZV, EBV, CMV: normal. MOG IgG: positive. Ig G: 3.9 mg/dl; protein: 40 mg/dl; Glucose: 60 mg/dl.	Anti-MOG encephalitis.	Brain MRI, CSF analysis.	None	Methylprednisolone, symptomatic treatment, etc.	Complete recovery
24	Fusco (43) (Spain)	1	F	30	RT-PCR	NM	Agitation, dysarthria and hallucinations.	aMRI: hyperintensities in diffuse left hemispheric.	Protein, glucose, cells, HSV-1: normal. Anti-NMDA: positive.	Anti-NMDA encephalitis.	Brain MRI, CSF analysis.	None	Methylprednisolone, symptomatic treatment, etc.	Partial recovery
25	Bhagat et al. (44) (USA)	1	M	54	RT-PCR	NM	Unconsciousness, generalized seizures.	aMRI: hyperintensities in the posterior aspect of right medial temporal lobe and para-hippocampal.	Protein: 108 mg/dl; glucose: 56 mg/dl; HSV-1, HSV-2: normal.	Limbic encephalitis.	Brain MRI.	AF, hypertension, glucose-6-phosphate dehydrogenase deficiency, and hepatosteatosis.	Lorazepam, levetiracetam, methylprednisolone, symptomatic treatment, etc.	Complete recovery
26	Ayatollahi et al. (45) (Iran)	1	F	18	RT-PCR	NM	Generalized tonic–clonic seizure.	aMRI: Claustrum FLAIR/T2 hyperintensities.	White blood cells: 20 cells/μl, no red blood cells, normal protein (30 mg/dl), glucose (41 mg/dl), and LDH (28 IU/L) levels.	Unknown Ab type of COVID-19-associated AE.	Brain MRI.	None	Methylprednisolone, antiepileptic drugs, symptomatic treatment, etc.	Partial recovery
27	Grimaldi et al. (46) (France)	1	M	72	RT-PCR	12 days	Bilateral upper-limb action tremor, a cerebellar syndrome, spontaneous diffuse myoclonus.	Brain PET with 18F-FDG: putaminal and cerebellum hypermetabolism associated with diffuse cortical hypometabolism.	Normal cell counts, a mildly elevated protein level (49 mg/dl).	Unknown Ab type of COVID-19-associated AE.	Brain PET.	None	Methylprednisolone, IVGI, symptomatic treatment, etc.	Complete recovery
28	Lee et al. (47) (Korea)	1	F	21	RT-PCR	NM	Abnormal behavior.	aMRI: unremarkable.	Protein: 402.4 mg/dl; glucose: 57.8 mg/dl. Anti-NMDA: positive.	Anti-NMDA encephalitis.	CSF analysis.	None	Methylprednisolone, IVGI, symptomatic treatment, etc.	Partial recovery
29	Mulder et al. (48) (USA)	1	M	40	RT-PCR	NM	Catatonia, autonomic instability.	aMRI: unremarkable	High red blood cell count, IL-6: 102.1 pg/ml; Protein: 838 mg/L.	Unknown Ab type of COVID-19-associated AE.	CSF analysis.	None	Plasmapheresis, symptomatic treatment, etc.	Complete recovery
30	Subramanyam et al. (49) (India)	1	M	47	RT-PCR	NM	Postural imbalance, difficulty walking, dysarthria, and hallucination.	aMRI: unremarkable	Protein, glucose, cell: normal. NMDAR, LGI1, AMPA12, and GABAb1-2: negative. Caspr2 and VGKC antibodies positive in serum.	Anti-Caspr2 encephalitis.	CSF analysis.	None	Methylprednisolone, IVGI, symptomatic treatment, etc.	Complete recovery
31	Zambreanu et al. (50) (UK)	1	F	66	RT-PCR	NM	Mental and behavioral disorders.	aMRI: hyperintensities in mesial temporal lobes and medial thalami.	Protein, glucose, fungal culture, HSV, VZV, EBV, CMV: normal.	Limbic encephalitis.	Brain MRI.	None	Methylprednisolone, IVGI, symptomatic treatment, etc.	Partial recovery
32	Serrano-Serrano et al. (51) (Spain)	1	M	70	RT-PCR	NM	Limb tremor with disturbance of consciousness.	aMRI: multiple disseminated hyperintensities.	Protein: 76 mg/dl. Glucose, cell: normal. HSV, HIV: negative.	Unknown Ab type of COVID-19-associated AE.	Brain MRI.	Hypertension, AF, COPD, HF	Methylprednisolone, symptomatic treatment, etc.	Complete recovery
33	Poorshiri et al. (52) (Iran)	1	M	1	RT-PCR	12 days	Generalized tonic-clonic seizures.	aMRI: hyperintensities in the entire corpus callosum, ventral aspect of medulla oblongata, and cerebellar vermis.	No white blood cells or red blood cells were seen. Protein: 108 mg/dl, Glucose: 72 mg/dl.	Unknown Ab type of COVID-19-associated AE.	Brain MRI.	None	Methylprednisolone, symptomatic treatment, etc.	Multiorgan damage
34	Shen et al. (53) (Singapore)	1	M	70	RT-PCR	13 days	Dysphasia and mild right sided weakness. Refractory status epilepticus.	aMRI: left mesial temporal lobe swelling with hyperintensity.	Protein: 60 mg/dl. Anti-GABA: positive.	Anti-GABA encephalitis.	Brain MRI, CSF analysis.	Diabetes, hypertension.	Methylprednisolone, symptomatic treatment, etc.	Complete recovery
35	McHattie et al. (54) (UK)	1	F	53	RT-PCR	NM	Palilalia, confusion, myalgia.	aMRI: hyperintensities in the left amygdala and anterior putamen.	Protein: 54 mg/dl. Glucose, cells: normal. Anti-NMDA: positive.	Anti-NMDA encephalitis.	Brain MRI, CSF analysis.	Breast carcinoma, depression, and psoriasis.	IVIG, hydroxychloroquine, symptomatic treatment, etc.	Complete recovery
36	Wang et al. (55)) (China)	1	M	68	RT-PCR	2 days	Unable to walk and with uroclepsia, coprolalia, and persecution delusion.	Brain CT scan: lacunar lesions in the left basal ganglia region.	COVID-19 IgM and IgG: positive.	Unknown Ab type of COVID-19-associated AE.	Brain MRI, CSF analysis.	Diabetes, hypertension.	IVIG, symptomatic treatment, etc.	Partial recovery
37	Bartley et al. (56) (USA)	3	NM	Mid-teens	RT-PCR	NM	➀ Subacute social withdrawal, insomnia, erratic behavior, paranoia-like fears, delusion, and paranoia. ➁ Foggy brain, word-finding difficulties, impaired concentration, and difficulty completing homework. ➂ Repetitive behaviors, anorexia, and insomnia.	➀ aMRI: white matter hyperintensities in the frontal lobes without enhancement. ➁ aMRI: unremarkable. ➂ aMRI: unremarkable.	➀ WBC: 4 (count,/μl); Protein: 117.0 (mg/dl); Ig G: positive. ➁ WBC: 1 (count,/μl); Protein: 80.0 (mg/dl); Ig G: positive. ➂ WBC: 1 (count,/μl); Protein: 19 (mg/dl); Ig G: negative; 3 unique oligoclonal bands.	➀ Unknown Ab type of COVID-19-associated AE. ➁ Unknown Ab type of COVID-19-associated AE. ➂ Unknown Ab type of COVID-19-associated AE.	➀ CSF analysis, Brain MRI. ➁ CSF analysis. ➂ CSF analysis.	➀ Marijuana use ➁ None ➂ None	➀ Methylprednisolone, IVIG, symptomatic treatment, etc. ➁ Methylprednisolone, IVIG, symptomatic treatment, etc. ➂ Lorazepam and olanzapine without immunotherapy	➀ Complete recovery ➁ Complete recovery ➂ Complete recovery

M, male; F, female; EBV, Epstein-Barr virus; TR-PCR, reverse transcription-polymerase chain reaction; HSV, herpes simplex virus; HCV, hepatitis C virus; HIV, human immunodeficiency virus; NMDA, N-Methyl-D aspartate; MOG, myelin oligodendrocyte glycoprotein; GABA, gamma aminobutyric acid; SARS-CoV-2, severe acute respiratory syndrome coronavirus 2; COVID, coronavirus disease; AMPA, a-amino-3-hydroxy-5-methyl-4-isoxazolepropionic acid; CRMP, collapsin response mediator protein; GAD, glutamic acid decarboxylase; aMRI, ordinary magnetic resonance imaging; eMRI, enhancement magnetic resonance imaging; CT, computed tomography; IL-6, interleukin-6; CSF, cerebrospinal fluid; IVIG, intravenous immunoglobulin; AF, atrial fibrillation; HF, heart failure; COPD, chronic obstructive pulmonary disease; AE, autoimmune encephalitis.

### 3.3. Neurological manifestations

**Figure 2:**
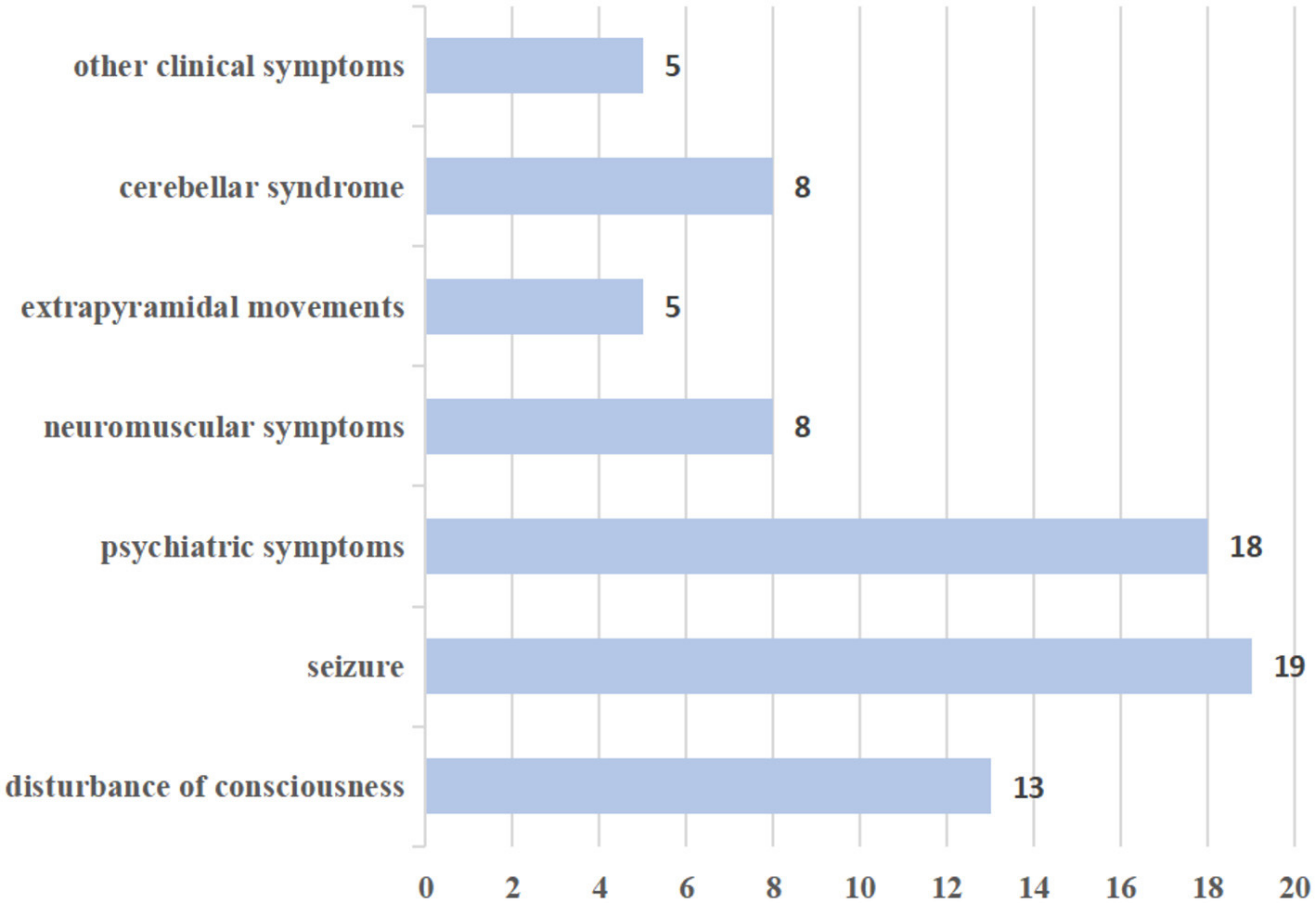
The frequency of various neurological manifestations.

Neurological manifestations in this study were reported in seven categories: (A) disturbance of consciousness; (B) seizures; (C) psychiatric symptoms; (D) neuromuscular symptoms such as myalgia, weakness, and myoclonus; (E) extrapyramidal movements; (F) cerebellar syndrome; and (G) other clinical symptoms. The frequency of various neurological manifestations is presented in Figure 2.

In this review, disturbances of consciousness were observed in 13 patients, primarily characterized by symptoms such as drowsiness, delirium, confusion, and unconsciousness. Additionally, 19 patients exhibited seizures, with 5 experiencing generalized tonic-clonic seizures and 2 experiencing refractory status epilepticus. In all, 18 patients in the study exhibited psychiatric symptoms, which mainly manifested as abnormal behavior, persecutory delusions, and auditory hallucinations. In addition, neuromuscular symptoms were observed, including myalgia, weakness, hypermyotonia, and myoclonus. Notably, many patients displayed extrapyramidal movements, such as choreiform movements in the hands and feet, peri-oral dyskinesias, bilateral upper-limb action tremors, and limb tremors. Eight patients had cerebellar syndrome, characterized by ataxia, dysarthria, and gait disturbance. Other clinical symptoms included ouroclepsia, palilalia, autonomic instability, headache, vertigo, and non-specific blurry vision.

### 3.4. Types of AE

**Figure 3:**
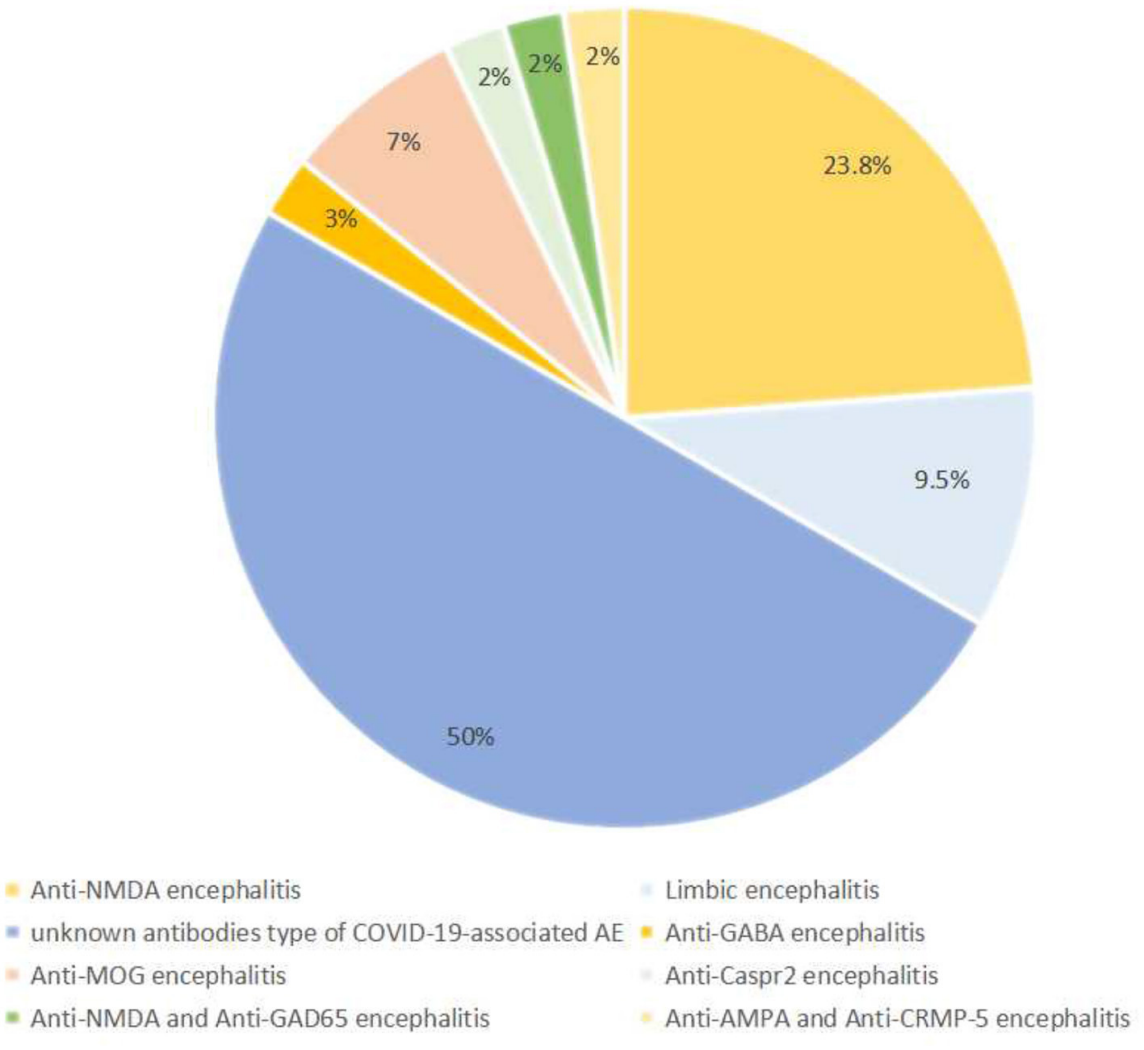
The frequency of various types of COVID-19-associated autoimmune encephalitis.

Among the studies included in this analysis, 42 patients were diagnosed with AE, including 21 (50%) cases of an unknown type of COVID-19-associated AE, 10 (23.8%) cases of anti-N-methyl-D aspartate (NMDA) encephalitis, 4 (9.5%) cases of limbic encephalitis, 3 (7.1%) cases of anti-myelin oligodendrocyte glycoprotein (MOG) encephalitis, 1 case of anti-gamma aminobutyric acid (GABA) encephalitis, 1 case of anti-Caspr 2 encephalitis, 1 case of anti-NMDA combined anti-glutamic acid decarboxylase-65 (GAD-65) encephalitis, and 1 case of anti-a-amino-3-hydroxy-5methyl-4-isoxazolepropionic acid (AMPA) combined anti-collapsin response mediator protein-5 (CRMP-5) encephalitis. The frequencies of the various types of AE are depicted in Figure 3.

In this study, the main type of COVID-19-associated AE is an unknown antibody (Ab) type of COVID-19-associated AE, where despite no detection of AE-related Ab in the CSF, a diagnosis of possible AE was made according to Graus criteria: first, they had an acute (< 2 weeks) or subacute (≤ 3 months) onset, accompanied by neurological and psychiatric symptoms or clinical syndrome signs. Second, they showed one or more of the following auxiliary examinations: CSF abnormalities, neuroimaging abnormalities, or electrophysiological abnormalities. Third, other potential causes were reasonably excluded (57). Notably, 21 patients were diagnosed with an unknown Ab type of COVID-19-associated AE; these patients exhibited consciousness disturbance, generalized tonic-clonic seizures, persecution delusion, autonomic instability, and dysarthria (Table 1) (24–27, 29–31, 39, 41, 45, 46, 48, 51, 52, 55, 56). The ages of these patients ranged from 1 to 88 years, with a mean age of 45.3 years. Among this group, 10 cases reported symptom onset ranging from 2 to 12 days (with an average of 8 days) from the positive SARS-CoV-2 RT-PCR.

In all, 10 studies included 10 patients diagnosed with anti-NMDA receptor encephalitis, ranging in age from 1 to 53 years (with an average of 24.2 years) (20–23, 28, 32, 33, 43, 47, 54). The neurological manifestations of these patients mainly included seizures, persecutory delusions, hallucinations, and abnormal behavior.

There were four cases of limbic encephalitis, with the main symptoms being generalized seizures and disturbances of consciousness (26, 35, 44, 50). Three cases reported anti-MOG encephalitis, and prominent clinical manifestations were vertigo, non-specific blurry vision, and assaultive behavior. Notably, the magnetic resonance imaging findings of anti-MOG encephalitis were usually diffuse and multifocal (36, 38, 42).

Additionally, four rare cases of AE were reported, including anti-Caspr 2 encephalitis, anti-NMDA combined anti-GAD65 encephalitis, anti-AMPA combined anti-CRMP-5 encephalitis, and anti-GABA encephalitis (37, 40, 49, 53). These patients mainly manifested hallucinations, postural imbalance, generalized seizures, and dysarthria.

### 3.5. Cerebrospinal fluid

Among 42 patients, 19 (45.23%) had elevated CSF protein levels, ranging from 49.0 to 838.0 mg/dl. Most of these cases showed mild elevation. The average glucose level in CSF was 81.7 mg/dl (range: 59.0–130.0 mg/dl), indicating raised levels. CSF glucose was found to be elevated in 9 (21.42%) patients, with a maximum value of 78 mg/dl. Additionally, four cases reported elevated levels of IgG in the CSF, and four cases reported the presence of oligoclonal bands.

### 3.6. MRI results

Among the 42 patients, 36 patients conducted ordinary MRI and 2 conducted enhanced MRI. Of these, 12 (33.33%) patients exhibited no significant abnormalities on ordinary MRI, while 24 (66.66%) patients displayed varying degrees of abnormalities. In the reviewed cases, hyperintensity has been observed in different regions, such as white matter, temporal lobe, parietal lobe, insular cortex, brainstem, cerebellum, and corpus callosum, and in some cases, it has been observed in multiple disseminated regions. Among 21 unknown types of COVID-19-associated AE patients, 12 (57.14%) patients exhibited abnormalities on ordinary MRI, which mainly manifested as hyperintensity in the frontal lobe, corpus callosum, and temporal lobe, and in severe cases, brainstem and multiple diffuse hyperintensities.

### 3.7. Medical history

The most frequently reported medical history was hypertension, accounting for 19.07% (*n* = 8), followed by diabetes, accounting for 9.52% (*n* = 4). Other medical history included autism spectrum disorders, migraine, kidney transplant, myasthenia gravis, glucose-6-phosphate dehydrogenase deficiency, and hepatosteatosis. Among the reviewed cases, in only three patients, COVID-19 vaccination was reported.

### 3.8. Outcome

Among the 42 patients, 29 (69.04%) achieved complete recovery, 8 (19.04%) showed partial recovery, and 1 experienced multiorgan damage, and in 2 cases the outcome was not reported. Two (4.76%) patients died due to sepsis and intracranial hemorrhage, respectively. Despite the varied symptoms of AE, most patients were able to fully recover or achieve partial recovery, with only a few experiencing residual neurological symptoms.

## 4. Discussion

AE is one of the neurological complications of COVID-19. This study conducted a review of case series and case reports to evaluate the neurological manifestations, types, and outcomes of COVID-19-associated AE. Previous studies have shown that in COVID-19-associated AE patients, the onset of symptoms can occur several weeks after or even during acute SARS-CoV-2 infection (55). In our review, we observed that the neurological manifestations of patients could be categorized into seven categories, including disturbance of consciousness, seizures, psychiatric symptoms, neuromuscular symptoms, extrapyramidal movements, cerebellar syndrome, and other clinical symptoms. The most common symptoms were seizures (19 cases), disturbance of consciousness (13 cases), and psychiatric symptoms (18 cases). COVID-19-associated AE patients who experienced epileptic seizures predominantly presented with generalized tonic-clonic seizures. COVID-19-associated AE can also manifest as psychiatric symptoms, such as cognitive decline, abnormal behavior, delusions of persecution, and hallucinations. Furthermore, some patients may initially experience neuromuscular symptoms or extrapyramidal movements, including myalgia, weakness, myoclonus, perioral dyskinesias, myoclonus-like movements, and limb tremors. COVID-19-associated AE patients also exhibited other clinical symptoms, including uroclepsia, palilalia, autonomic instability, headache, vertigo, and non-specific blurry vision.

In this review, we identified several types of COVID-19-associated AE, including an unknown type of COVID-19-associated AE, anti-NMDA encephalitis, limbic encephalitis, anti-MOG encephalitis, anti-GABA encephalitis, anti-Caspr-2 encephalitis, anti-NMDA combined anti-GAD-65 encephalitis, and anti-AMPA combined anti-CRMP-5 encephalitis. In our cohort of patients, the most frequently reported COVID-19-associated AE was an unknown Ab type of COVID-19-associated AE (50%), followed by anti-NMDA encephalitis (23.8%), limbic encephalitis (9.5%), and anti-MOG encephalitis (7.1%). Additionally, rare cases of AE were also observed, such as anti-Caspr 2 encephalitis, anti-NMDA combined anti-GAD-65 encephalitis, and anti-AMPA combined anti-CRMP-5 encephalitis.

The mechanism of COVID-19-associated AE is extremely complex and not yet fully understood. One theory suggested that the occurrence of COVID-19-associated AE may be attributed to molecular mimicking between viral proteins and neuronal self-antigens (58). Additionally, it has been observed that severe COVID-19 patients exhibit higher levels of inflammatory cytokines compared to non-severe patients (33, 59). In the case of anti-NMDAR encephalitis, elevated levels of interleukin-6 (IL-6) are commonly found during the inflammatory phase of the disease, which further stimulates the production of autoantibodies (32, 33). Moreover, SARS-CoV-2 infection triggers an inflammatory response in the central nervous system, similar to other pathogens. This immune response involves the activation of neutrophils, monocytes, macrophages, dendritic cells, and natural killer (NK) cells, leading to the release of numerous inflammatory factors (60).

The diagnosis of AE is usually based on a combination of clinical presentation, imaging analysis, CSF analysis, and electroencephalogram (61). In AE patients, MRI scans can detect abnormalities in approximately 60% of cases, while 40% of scans show no significant changes. Among patients with positive MRI findings, most exhibit T2 and Flair high signal shadows on one or both sides of the medial temporal lobe (including the hippocampus and sulcus gyrus), insular lobe, and amygdala (62). These abnormalities may appear progressively but often disappear after immunotherapy. CSF analysis typically lacks specificity, with approximately 30% of results being normal (60, 61). A retrospective multicenter study suggested that 72% of patients did not fulfill AE diagnostic criteria, resulting in a high proportion of mistaken for AE (62). It is worth considering that during the initial phase of the pandemic, the high viral circulation and incidence in the general population may have led to mistakenly associating AEs with COVID-19, resulting in a misinterpretation. The reasons for misdiagnosis may be related to overinterpretation of positive serum antibodies and misinterpretation of functional/psychiatric or non-specific cognitive dysfunction such as encephalopathy (62).

Furthermore, little emphasis has been placed on distinguishing between delirium and seizures caused by metabolic derangements in this review. Metabolic encephalopathy (ME) arises from local or global brain edema, neurotransmitter transmission disorders, and the accumulation of metabolic toxins due to damage to the blood-brain barrier, free radical damage, and apoptosis. It can manifest as coma, hemiplegia, epilepsy, and other symptoms (63). When considering ME, medical history, relevant laboratory tests, and imaging analysis need to be fully integrated. Also, when considering AE, physicians need to differentiate it from ME.

Regarding treatment strategies and outcomes, almost all patients received methylprednisolone, intravenous immunoglobulin, plasmapheresis, and oral prednisone therapy. In our study, we found that despite the potentially life-threatening risks of COVID-19-associated AE, most patients completely or partially recovered, with a few having residual symptoms of neurological damage.

This study has certain limitations. First, this systematic review includes only case reports and case series. In addition, many poor-quality reports have been published during the first stage of the health emergency, and the data are frequently partial or biased in consideration of the high circulation of the virus. Therefore, publication bias is inevitable. Second, we must admit that there are flaws in the inclusion criteria for case and case series reports. Among 42 COVID-19-associated AE patients, 39 (92.85%) cases were RT-PCR positive, and 3 (7.14%) cases were positive for SARS-CoV-2 serum antibody. This discrepancy might introduce bias since not all COVID-19-associated AE patients demonstrated positive RT-PCR either at the onset of AE symptoms or after SARS-CoV-2 infection. Third, most case reports and case series lack detailed descriptions of COVID-19 vaccination. This may lead to detection bias as some diagnoses rely on serum antibodies. Fourth, since the search was limited to articles published in English, some relevant articles in other languages were omitted. Finally, most studies did not have sufficient data, such as the time from onset to recovery and vaccination against COVID-19.

## 5. Conclusion

This systematic review provides a comprehensive summary of the neurological manifestations, types, and outcomes in patients who developed AE as a complication of COVID-19. The main type of COVID-19-associated AE identified in this study is an unknown Ab type of COVID-19-associated AE. Although COVID-19-associated AE can present potentially life-threatening risks, the majority of patients survived, with some patients reporting residual neurological symptoms.

## Data availability statement

The original contributions presented in the study are included in the article/supplementary material, further inquiries can be directed to the corresponding author.

## Author contributions

HX and LZ carried out the conception and design of the research, and HX and DX participated in the acquisition of data. HX carried out the analysis and interpretation of data. HX and HH drafted the manuscript and DX participated in the revision of the manuscript for important intellectual content. All authors read and approved the final manuscript.
